# A Quantitative Analysis of Cold Water for Human Consumption in Hospitals in Spain

**DOI:** 10.1155/2016/6534823

**Published:** 2016-06-16

**Authors:** A. G. González, J. García-Sanz-Calcedo, D. R. Salgado, A. Mena

**Affiliations:** ^1^School of Design Engineering, Department of Mechanical, Energy, and Materials Engineering, University of Extremadura, 06800 Mérida, Spain; ^2^Department of Projects, University of Extremadura, 06007 Badajoz, Spain; ^3^School of Industrial Engineering, Department of Mechanical, Energy, and Materials Engineering, University of Extremadura, 06007 Badajoz, Spain; ^4^School of Industrial Engineering, Department of Engineering Design and Projects, University of Huelva, 21003 Huelva, Spain

## Abstract

An estimation of the water used for human consumption in hospitals is essential to determine possible savings and to fix criteria to improve the design of new water consumption models. The present work reports on cold water for human consumption (CWHC) in hospitals in Spain and determines the possible savings. In the period of 2005–2012, 80 Eco-Management and Audit Schemes (EMAS) from 20 hospitals were analysed. The results conclude that the average annual consumption of CWHC is 1.59 m^3^/m^2^ (with a standard deviation of 0.48 m^3^/m^2^), 195.85 m^3^/bed (standard deviation 70.07 m^3^/bed), or 53.69 m^3^/worker (standard deviation 16.64 m^3^/worker). The results demonstrate the possibility of saving 5,600,000 m^3^ of water per year. Assuming the cost of water as approximately 1.22 €/m^3^, annual savings are estimated as 6,832,000 €. Furthermore, 2,912 MWh of energy could be saved, and the emission of 22,400 annual tonnes of CO_2_ into the atmosphere could be avoided.

## 1. Introduction

The hospital is a tertiary sector building in which, due to the nature of its usage, a large number of human resources are needed. The objectives outlined by the European Council in March 2007 were initially to reduce the total energy consumption by 20% (based on the 2005 consumption) and to cut greenhouse emissions by 20% to below the emissions recorded in 1990. These objectives were designed to reduce the use of resources [1].

Although the quantity of water available on Earth today is sufficient to cover the needs of the population, continual inappropriate and excessive usage could lead to a lack of resources within a few years. To overcome this situation, a change in current consumption rates is crucial. This would focus on (i) the preservation of water and an improvement in water management, (ii) encouragement of a greater respect and sensitivity towards the usage of water, and (iii) a more balanced distribution and emphasis on its ecological and social value [2].

In Spain, annual water consumption in cities totals 5,000,000,000 m^3^, which is 20% of the country's total consumption. The average daily usage is 171 litres per person, at a cost between 0.91 €/m^3^ and 1.69 €/m^3^ depending on the region [3].

At European Union level, the Water Framework Directive sets objectives to be achieved by all member states, but water quantities are relatively less considered than water quality, and achieving the goals often requires collaboration as water is a shared resource that requires a holistic and integrated approach [4].

The Pacific Institute for Studies in Development, Environment and Security highlights the importance of the hospitals in the construction sector, for its usage of large quantities of water in most procedures [5]. The areas where water consumption in a hospital is high are as follows: patients rooms 20%, domestic hot water (DHW) 15%, laundry areas 15%, maintenance of green areas 10%, therapeutic pools 9%, kitchens 8%, cleaning 5%, refrigeration towers 5%, sterilization 5%, heating ventilation and air conditioning (HVAC) 4%, and others 4% [6].

The typical ratios of usage across European hospitals indicate an average annual consumption between 182.5 and 365 m^3^ per bed [7]. However, these indicators fluctuate greatly depending on the locations under study, the type of establishment, the date of construction, the number of users, the number of workers, and the possible green areas it may have. In the USA this consumption ranges from 109.5 to 552.61 m^3^/bed [8–10] and in the UK from 193.45 to 415.37 m^3^/bed [11] and in Germany, as reported in some studies, it lies in the range 109.5–223.02 m^3^/bed [12], reaching a maximum of 247.84 m^3^/bed [13]. Canadian studies reveal even higher ratios between 328.5 and 657 m^3^/bed [14]. Other examples of these figures come from the Mexican Institute of Water Technology, which reports annual consumption as 292 m^3^/bed [15], whereas the Pan-American Health Organization (PAHO) indicates 164.25 m^3^/bed [16].

**Figure 1:**
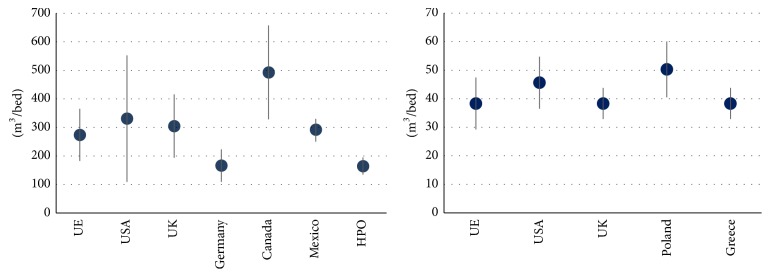
Annual average consumption of CWHC and DHW in healthcare centres per hospital bed.

To assess hot water consumption, a series of studies was carried out. These studies show that the annual average per hospital bed varies between 29.2 m^3^/bed and 47.45 m^3^/bed in Europe [17] and between 36.5 and 54.75 m^3^/bed in the USA [18]. In Greece, this figure ranges between 32.85 and 43.8 m^3^/bed [19, 20]. Bujak [21] estimated that the average annual consumption of hot water in a hospital lies between 40.52 and 60.05 m^3^/bed.  Figure 1 shows the values of annual average consumption ratios of CWHC and DHW per hospital bed.

Though some studies related to the management of water consumption in Spain have been undertaken [22], methods for saving water have not yet been studied in a systematic form, even though expectations of saving water are high. This information is based on studies of 903 hospitals, which were operative in 2013, and extends to a total of 163,585 beds. However, the potential energy saving in hospitals has elsewhere been studied [23].

The small amount of research, which has been done up until now, has only been carried out on a small number of sample buildings and therefore has little statistical relevance. The purpose of the present work is to analyse and assess the consumption of CWHC in hospitals in Spain, depending on different variables, and to estimate the possibilities of savings.

## 2. Methodology

To obtain valuable data with high statistical significance in the results, an analytical study was performed between 2005 and 2012 in 20 Spanish hospitals, which had been built between 1980 and 2005.

Data was collected and analysed according to the regulations of EMAS [24], a voluntary environmental management instrument which recognises those organizations which not only have set up an environmental management system [25] but also have reached an agreement of continual improvement, which is verified through independent audits [26, 27]. EMAS is a management tool developed for companies and other organizations, to evaluate, inform, and improve their environmental achievements. 80 EMAS statements have been analysed in hospitals [28].

The consumption of CWHC in 20 hospitals was examined between 2005 and 2012.  Table 1 shows the list of particular hospitals under study. A reduction factor for the consumption of CWHC has been applied in the case of hospitals with gardening areas (reduction factor 10%) or laundry facilities (reduction factor 15%) [29, 30].

The figures for the number of beds and the number of workers analysed by the EMAS were obtained from annual data published by the Ministry of Health [31]. In both cases the figures were acquired by calculating the average in relation to the range of years. In order to calculate the area of the hospital, only the built surface area (m^2^) of the facilities has been taken into consideration.

In the present study, two different analyses were conducted. Firstly, an analysis of three water consumption indicators was carried out, namely, the average annual water consumption in relation to built surface area (floor area), the number of workers, and the number of beds. The second analysis was conducted in order to obtain more detailed results from the statistical data used in this research, for which analysis of variance (ANOVA) tests were carried out using the factors presented in Table 2. In this sense, it is important to note that an ANOVA test requires all samples to follow a normal distribution and to have the same variance. To prove that these indicators verify a normal distribution, the Levene test (an inferential statistic used to assess the equality of variances for a variable calculated for two or more groups) [32] was used. ANOVA is a statistical tool used to determine whether there are any significant differences between the means of three or more unrelated groups of data. In particular, ANOVA compares the means between the groups and determines whether any of those means are significantly different from each other.

The Gross Domestic Product (GDP) is the monetary value of all finished goods and services produced within a country's borders during a specific period of time, and it is considered a representative indicator that measures the growth or decrease of goods and services production. GDP is one of the primary measures used by decision-makers and financial and other institutions to evaluate the health of the economy. An increase in real GDP is interpreted as a sign that the economy is doing well, while a decrease indicates that the economy is not working at its full capacity. Real GDP is linked to other macro-economic variables such as employment, economic cycles, productivity, and long-term economic growth. In this sense, it is reasonable to consider that the GDP can be related to the services offered by hospitals and that such services could directly be related to the water consumption. The GDP has been divided into four ranges in this study (Table 2).

Heating degrees-day year (HDDY) is defined as the sum of the difference between a reference temperature and the average temperature of the day (taking into account all the days in a period) when such a temperature is lower than 15°C:

(1) HDDY C°=∑i=1n15−Tmax+Tmin2·Xc,

where *T*_max_ and *T*_min_ represent the maximum and the minimum daily temperature, respectively, and *X*_*c*_ is a logical coefficient that will equal unity when the average daily temperature is lower than 15°C and zero for values exceeding 15°C.

## 3. Results

In this section, firstly, the analysis of the correlation between the average annual consumption of water and the three indicators considered (built surface area, number of workers, and number of beds) is presented. Secondly, the ANOVA tests according to the factors listed in Table 2 are presented. The following results are obtained from the water consumption data given in Table 1.

### 3.1. Correlations between Average Annual Consumption of CWHC and Built Surface Area, Number of Workers, and Number of Beds

All possible correlations were accounted for to further conclude that a linear dependence is that which best describes the sample behaviour. This is in good agreement with some studies on hospital management elsewhere reported [33], which modelled the correlation among built surface area, number of workers, and number of beds.

#### 3.1.1. Relation between Average Annual Consumption of CWHC and the Built Surface Area (*A*)

**Figure 2:**
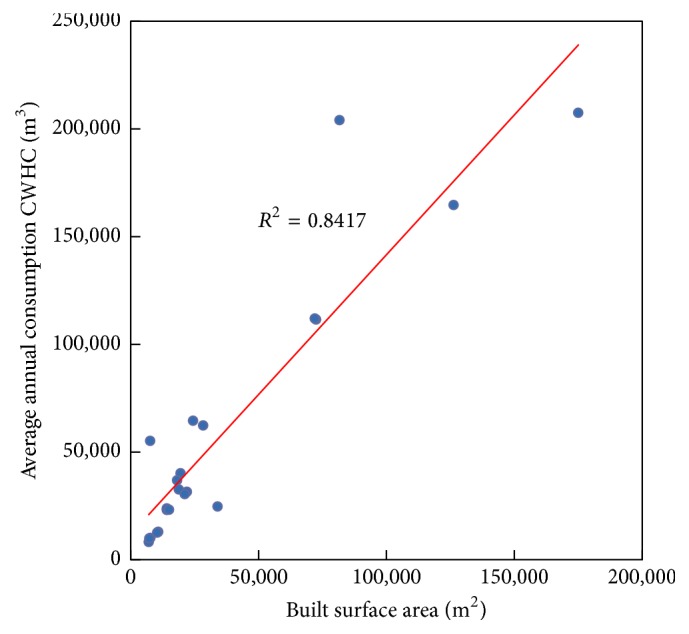
Relation between the average annual consumption of CWHC and the built surface area in a hospital.

The relation between consumption of CWHC and the built surface area of a hospital is shown in Figure 2, which indicates a good correlation factor (*R* ^2^ = 0.8417).

Equation (2) shows the mathematical expression for the linear fit in Figure 1:

(2) $$\begin{array}{c} \text{WC}=1.30A+\text{11,791}, \end{array}$$

where WC represents the average annual consumption of CWHC in m^3^ and *A* the built surface area in m^2^ of a hospital, respectively.

#### 3.1.2. Relation between the Average Annual Consumption of CWHC and the Number of Workers (NW)

**Figure 3:**
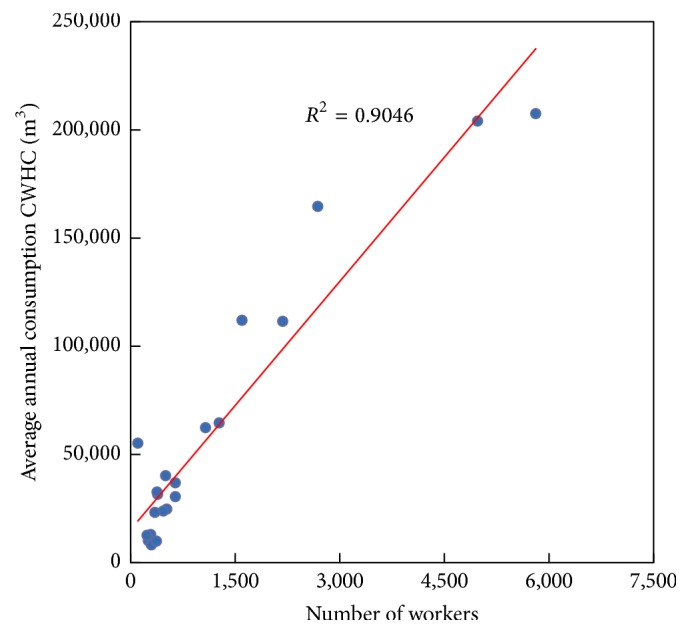
Relation between the average annual consumption of CWHC and the number of workers in a hospital.

In this case, the correlation factor (*R* ^2^ = 0.9046) shows a higher relation than in the above case.  Figure 3 shows the correlation between the average annual consumption of CWHC and the number of workers in a hospital, and (3) sets the mathematical expression for the corresponding linear fit:

(3) $$\begin{array}{c} \text{WC}=38.26\text{NW}+\text{15,221}, \end{array}$$

where WC represents the average annual consumption of CWHC in m^3^ and NW the number of workers in a hospital, respectively.

#### 3.1.3. Relation between the Annual Average Consumption of CWHC and the Number of Beds (NB)

**Figure 4:**
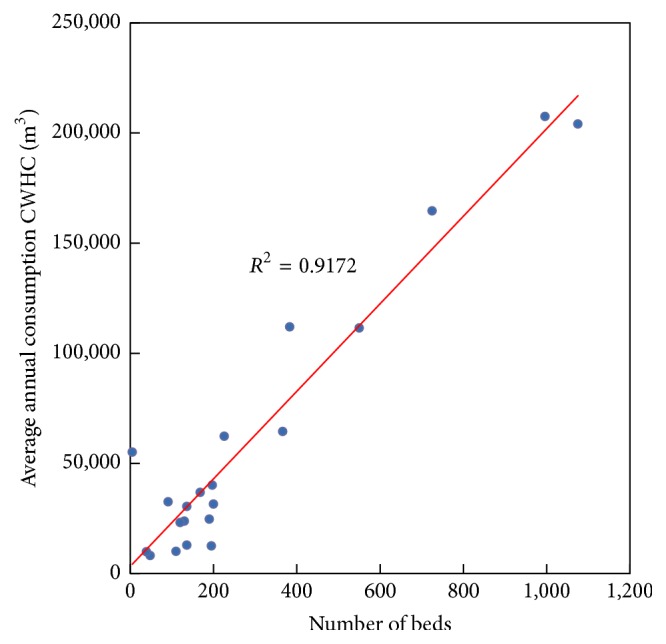
Relation between the average annual consumption of CWHC and the number of beds in a hospital.

Finally, the plot and the regression expression for the relation between the average annual consumption of CWHC (in m^3^) and the number of beds (NB) are indicated in Figure 4 and in (4), respectively. Note the correlation coefficient (*R* ^2^ = 0.9172) is the highest for the three analysed indicators to estimate water consumption in a hospital:

(4) $$\begin{array}{c} \text{WC}=198.77\text{NB}+\text{3,284}, \end{array}$$

where WC represents the average annual consumption of CWHC in m^3^ and NB the number of beds in a hospital.

### 3.2. ANOVA Results

In this subsection, the results obtained from the statistical analysis of variance (ANOVA) are presented. The factors listed in Table 2 as well as the ratios between the average annual consumption of CWHC in a Spanish hospital and the three abovementioned water consumption indicators (built surface area, number of workers, and number of beds in a hospital, resp.) are next analysed.  Table 3 lists the obtained *p* values in the analysis of variance. The null hypothesis in ANOVA test states that the population means for all conditions are the same. In order to determine whether any of the differences between the means are statistically significant, the *p* value should be compared to the significance level to assess the null hypothesis. A significance level of 0.05 (denoted by *α*) is assumed for the present study, provided such value is regarded to perform appropriately. If the *p* value is less than or equal to the significance level, that is, *p* value ≤ 0.05, the null hypothesis can be rejected and it could be concluded that not all of population means are equal. Otherwise, if the *p* value is greater than the significance level, there is not enough evidence to reject the null hypothesis that the population means are all equal.

#### 3.2.1. Water Consumption as Related to the Type of Management (TM)

Considering the type of management in a hospital as a factor, the results of ANOVA test present significant differences (*p* < 0.05) between the average annual water consumption in a hospital and one of three indicators used, namely, the indicator related to the number of beds, where great differences (*p* = 0.03) have been observed, as shown in Table 3. The *p* value is widely used in statistical hypothesis testing, specifically in null hypothesis significance testing. In statistical studies, one first chooses a model (the null hypothesis) and a threshold value for *p*, called the significance level of the test, traditionally 1% or 5%, and denoted as *α*. If the *p* value is less than or equal to the chosen significance level (*α*), the test suggests that the observed data is inconsistent with the null hypothesis, and so the null hypothesis must be rejected. For typical analysis, using the standard *α* = 0.05 cutoff, a widely used interpretation is that a small *p* value (≤0.05) indicates strong evidence against the null hypothesis, so it is rejected; and a large *p* value (>0.05) indicates weak evidence against the null hypothesis (fail to reject). For the particular case of type of management (TM), Table 3 shows that *p* < 0.05 only for the indicator accounting for the number of beds, which means that there is a strong evidence against the null hypothesis.

#### 3.2.2. Water Consumption as Related to Gross Domestic Product (GDP)

The results from the ANOVA test considering the GDP as a factor show great differences in the three statistical indicators as can be observed in Table 3. In other words, it can be concluded that there is no direct GDP link with the consumption of water according to the area, the number of workers, nor the number of beds.

#### 3.2.3. Water Consumption as Related to Heating Degrees-Day Year (HDDY)

Taking into account the HDDY factor, the outcome of the test shows differences in one of the three indicators, namely, the indicator of the number of beds (*p* = 0.03). However, there is no evidence of variance for the indicator of the area (*p* = 0.39) and for that of the number of workers (*p* = 0.27). Thus, there is no direct relationship between HDDY and the water consumption in hospitals in Spain according to the area and number of workers. There is however a link between HDDY and the number of beds.

#### 3.2.4. Water Consumption as Related to the Hospital Categorization in terms of the Number of Beds (HCNB)

The analysis of variance considering the category of the hospital as a factor (Table 2) shows great differences among the three statistical indicators, specifically that related to the built surface area of the hospital (Table 3).

Due to the existence of these substantial differences, the Fisher test was carried out in order to thoroughly examine these differences, and it proves that there is no direct link between the category of a hospital (HCNB) and the water consumption in relation to the number of workers or beds (Table 4). There is, however, a link between the HCBN and the built surface area of the hospital. The Fisher test is a statistical significance test used to compare sample means and is proved to be valid for any sample size.

Table 4 lists data corresponding to the analysis of the sample means of various hospital types, according to their HCNB factor. In particular, mean diff. stands for the difference between the means of the two compared samples in each row. The standard error of the mean (SEM) is a measure of how far a particular sample mean is likely to be from the true population mean and is always smaller than the standard deviation (SD). All other terms (*t*-value, prob., and Sig.) allow evaluation of the degree of similarity between the means of the samples compared. Finally, the lower and upper confidence limits (LCL and UCL) define the 95% confidence interval for the true mean difference between the means.

#### 3.2.5. Water Consumption as Related to Geographic Location (GL)

The results collected when considering location as a factor show significant differences in the average annual water consumption of CWHC in a hospital in relation to the number of beds (*p* = 0.01) and no statistical significance according to the built area of the hospital (*p* = 0.71) nor the number of workers (*p* = 0.36). Therefore, it can be concluded that there is a direct relationship between the water consumption based on its location and the number of beds in a hospital.

#### 3.2.6. Water Consumption as Related to the Range of Years

According to the results, the consumption of water according to the number of beds and workers and built area of the hospitals, one of the main explanations of such a significant reduction in CWHC between 2005 and 2007 is the impact of the sensitization and awareness campaigns about water savings. ANOVA test was carried out in order to show the influence of this campaign by using the average consumption of water as a main factor between the abovementioned years and then again from 2007 until 2012. Substantial differences were noted in one of the three indicators, namely, the statistical significance (Table 3) in the indicator related to the built surface area (*p* = 0.03), though there is no such a link for the number of workers (*p* = 0.12) nor for the number of beds (*p* = 0.23). Therefore, taking into account the water consumption, there is no direct link with the number of workers nor with the number of beds. There is, however, a direct relation to the built surface area of the hospital.

**Figure 5:**
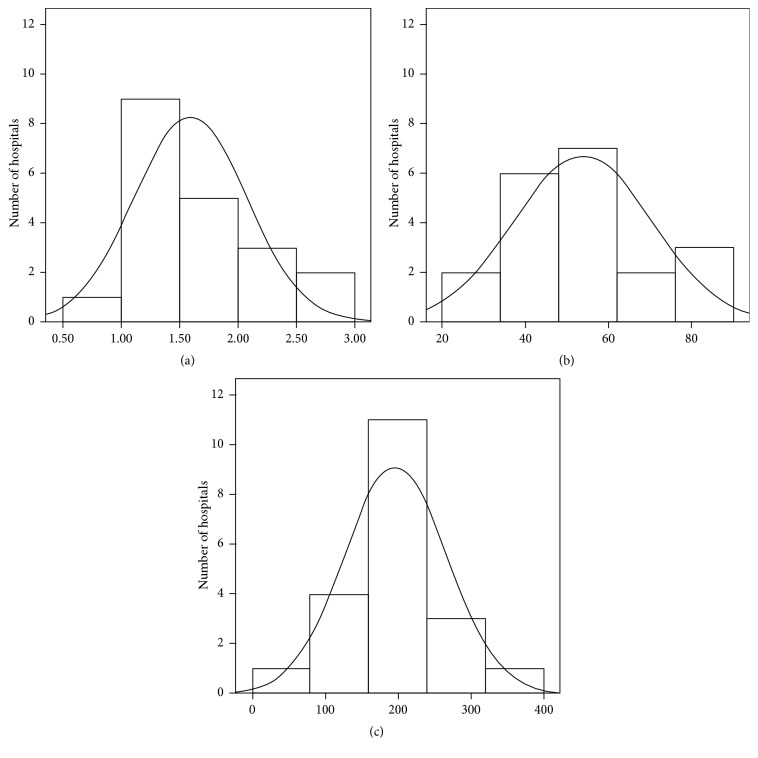
Average consumption in m^3^ of CWHC for each indicator: (a) built surface area, (b) number of workers, and (c) number of beds.

The final ratio of this study is 195.85 m^3^/year/bed. Significant consumption averages are shown in Figure 5 according to the statistical indicators.  Table 5 shows the classification according to percentiles and type of statistic indicator.

If 34.24 m^3^ water per hospital bed is assumed as average saving and 163,585 beds are taken to be available nationwide by the time the present study was carried out, the above results yield a potential annual water saving in Spanish hospitals of 5,600,000 m^3^. This implies an annual saving of 6,832,000 € if the cost of water is assumed as 1.22 €/m^3^. Additionally it is possible to achieve an energy saving of 2,912 MWh, which would avoid the emission of 22,400 tonnes of CO_2_ into the atmosphere every year. To work out this saving, an atmospheric emission of 4 kg of CO_2_ for every m^3^ of water has been calculated, which accounts for the emissions due to impulsion, purification, and depuration and for an energetic intensity of 0.52 kWh/m^3^ [34].

The indicators listed in Table 6 have been acquired through the investigation of the averages of different analysed EMAS.

## 4. Discussion

Any action to improve the efficiency of a hospital ought to account for both the climatic and working conditions in this kind of building. It must not disregard other requirements, for instance, the accessibility, safety, and reliability of its facilities.

Another factor that has to be analysed is the presence of* Legionella*, which is usually present in cooling towers and hydrological networks and in the production equipment of domestic hot water, mainly in accumulation stores where the stratification conditions boost its proliferation [35]. Interruptions in the water supply may create, in any section of the hydrological network, the conditions required for the bacteria to thrive and thus pollute the water once the supply is restored. In addition to this, sand and dust contain inactive forms of* Legionella*, which can move through the air and then plant themselves, thus polluting the cooling tower collectors. On one hand, purges in the facilities contribute to a reduction in the risk of this bacteria spreading but on the other hand, the water consumption level greatly increases.

An important element of the saving of water has been seen to be directly linked to the daily management of a hospital [36], in which it is possible to directly control the water consumption associated with both workers and users. Therefore it is suggested that workers increase their awareness of the importance of saving water through additional training and campaigns to optimise the sensitivity surrounding water saving and the rational use of water. The rain must also be used advantageously; it can be collected from the roof and used to irrigate green areas. This is an option, which would reduce the consumption of CWHC and consequently its environmental impact. The storage of this water, however, is not recommended. Another advisable strategy is to use specially constructed wells for the watering of green areas. Greywater, which comes from showers and sinks, must not be reused under any circumstances in this kind of building, as aseptic conditions take priority.

Measures for energy saving in hospital management should mainly focus on both domestic hot water savings and the increase of energy efficiency in production installations, given that such facilities are typically linked to high rates of energy consumption. However, efforts should also be made to account for the exploitation, channelling, and recovery of water from cooling/condensation towers and for the installation of electronic counters to monitor consumption rates and potential leaks.

The installation of atomizers, specific saving devices to be screwed in taps and showers, is also recommended. Air is injected to the water flow so that the speed of the flow stream is increased and the flow rate is thus reduced. Even though atomizers apparently increase the flow rate, water savings associated with their use are reported to range between 30% and 50%.

With regard to gardening, savings of around 25–30% can be achieved by landscape adaptation of the surroundings through xeriscape techniques, by the selection of native species, and by the use of efficient and programmable irrigation systems.

The use of floor cleaning devices based on microfiber fabric in hospitals is proved to reduce water consumption as well as decrease the needs for chemicals.

In all cases ISO-14000 and EMAS certifications are suggested procedures for improved management of hospital infrastructures, as can be seen from different ISO-14000 studies [37] and EMAS studies [38]. Being in possession of such certificates implies a greater ability to implement the improvement of the hospital image and environmental surroundings. At the same time, environmentally speaking, wastage and how it is discharged cannot be overlooked nor omitted.

The information related to the environmental efficiency of Spanish hospitals registered in EMAS is sufficient but there are certain deficiencies in the indicators (surface built area, number of beds, and number of workers) that make it difficult to make a comparative evaluation. This is because the chosen indicators are not always used with the same criteria, and consequently they do not quantify the analysed parameter appropriately. In turn, this is likely to be because of a wrongly chosen indicator. There are studies which indicate such deficiencies according to the used indicators, like the EVER study [39].

The results of this research can be useful to quantify the exact cost of water consumption. It could be interesting to repeat the same study in different organizations and hospitals in other countries, in order to establish some comparisons.

The results are extrapolated to similar buildings with limitations due to the wide variety of healthcare building designs mainly based on architectural conception, climate conditions, interior facilities, and building locations.

## 5. Conclusion

It can be concluded that there is a link between the cold water for human consumption (CWHC) in hospitals and the built surface area of the hospital (*A*) and the number of beds (NB). However, the number of workers (NW) has no significant statistical relation to such consumption.

Furthermore, it has been proved that the factors based on the hospital category depending on number of beds (HCNB), type of management (TM), heating degrees-day year (HDDY), and geographic location (GL) have a direct relationship with water consumption. There is no such link regarding the GDP.

The statistical indicator of the number of workers (NW) is not considered appropriate to be used as a ratio to quantify the consumption of water. This indicator is the most used in EMAS and it has been proved throughout this study that it is not consistent enough and it is not adequately quantified.

## Figures and Tables

**Table 1:** List of hospitals under study.

Hospital	Management	Area (m^2^)	Number of workers	Number of beds	CWHC (m^3^/year)	Province
Hospital Asepeyo de Coslada	Private	22,000	389	200	31,536	Madrid
HM Universitario de Madrid	Private	7,717	257	110	10,074	Madrid
HM Universitario Montepríncipe	Private	19,521	503	197	40,147	Madrid
HM Universitario Torrelodones	Private	10,808	291	136	12,928	Madrid
HM Universitario Sanchinarro	Private	33,989	520	190	24,692	Madrid
Hospital Clínico San Carlos	Public	175,000	5,811	996	271,270	Madrid
Hospital Juan Ramón Jiménez	Public	126,241	2,685	725	215,232	Huelva
Hospital Costa del Sol	Public	24,408	1,271	366	71,690	Málaga
HAR de Benalmádena	Public	7,077	300	48	8,184	Málaga
Hospital Virgen de las Nieves	Public	42,734	4,977	1,075	266,767	Granada
Hospital Victoria Eugenia	Private	7,330	372	39	9,889	Sevilla
Hospital General de Valencia	Public	18,209	2,184	550	145,773	Valencia
Fundación Hospital Calahorra	Public	6,683	382	91	36,195	La Rioja
Hospital Galdakao-Usansolo	Public	72,000	1,599	383	131,730	Vizcaya
Hospital de Zumárraga	Public	14,125	470	130	23,801	Guipúzcoa
Hospital Asepeyo Sant Cugat	Private	15,000	350	120	23,194	Barcelona
Hospital de Figueres	Private	18,186	643	168	36,857	Gerona
Hospital de Manacor	Public	28,333	1,076	226	62,330	Baleares
Hospital de Palamós	Private	21,151	643	136	30,455	Gerona
Hospital Perpetuo Socorro	Private	10,409	237	195	12,568	Las Palmas

**Table 2:** Classification of the factors considered in the statistical analysis of the collected data.

Factors	Distribution regarding factors
Type of management (TM)	Public
	Private

Gross Domestic Product (GDP)	GDP 1: <20,000 €
	GDP 2: 20,000 €–25,000 €
	GDP 3: 25,000 €–30,000 €
	GDP 4: >30,000 €

Heating degrees-day year (HDDY)	HDDY 1: 0° to 250°C
	HDDY 2: 250° to 500°C
	HDDY 3: 500° to 750°C
	HDDY 4: 750° to 1000°C
	HDDY 5: 1,000° to 1,250°C
	HDDY 6: 1,250° to 1,500°C
	HDDY 7: >1,500°C

Hospital category depending on the number of beds (HCNB)	HCNB 1: <200 beds
	HCNB 2: 200 to 500 beds
	HCNB 3: 500 to 1,000 beds
	HCNB 4: >1,000 beds

Geographic location (GL)	Madrid
	Andalucía
	Valencia
	Rioja
	País Vasco
	Cataluña
	Canarias

Range of years	2005–2007
	2008–2012

**Table 3:** Analyses of variance.

Test factors	Consumption ratios		
	$\frac{m^{3}\;\;average\;\;water\;\;consumption}{m^{2}\;\;built\;\;\;surface\;\;area}$	$\frac{m^{3}\;\;average\;\;water\;\;consumption}{Number\;\;of\;\;workers}$	$\frac{m^{3}\;\;average\;\;water\;\;consumption}{Number\;\;of\;\;beds}$
Type of management (TM)	*p* = 0.14	*p* = 0.88	*p* = 0.03^*∗*^
Gross Domestic Prod. (GDP)	*p* = 0.52	*p* = 0.27	*p* = 0.23
Heating degrees-day year (HDDY)	*p* = 0.39	*p* = 0.27	*p* = 0.03^*∗*^
Hospital categories (HCNB)	*p* = 0.01^*∗*^	*p* = 0.79	*p* = 0.51
Geographical location (GL)	*p* = 0.71	*p* = 0.36	*p* = 0.01^*∗*^
Range of years (2005–2007 and 2008–2012)	*p* = 0.03^*∗*^	*p* = 0.12	*p* = 0.23

^*∗*^At the 0.05 level, the population means are significantly different.

**Table 4:** Fischer test for means comparison with 0.05 of significance level.

HCNB	Mean diff.	SEM	*t-*value	Prob.	Sig.	LCL	UCL
3-1	−0.11	0.24	−0.45	0.66	0	−0.62	0.40
2-1	0.68	0.24	2.83	0.01	1	0.17	1.19
2-3	0.79	0.31	2.57	0.02	1	0.14	1.44
4-1	1.05	0.39	2.68	0.02	1	0.22	1.88
4-3	1.16	0.43	2.66	0.02	1	0.23	2.08
4-2	0.37	0.43	0.84	0.41	0	0.56	1.29

**Table 5:** Classification according to percentiles and type of statistic indicator.

Indicator	Average annual consumption in m^3^ of CWHC					
	Percentiles					
	10%	25%	50%	75%	90%	Average
$\frac{Average\;\;water\;\;consumption\;\;{(m}^{3})}{Built\;\;surface\;\;area\;\;{(m}^{2})}$	1.18	1.28	1.49	1.80	2.23	1.59
$\frac{Average\;\;water\;\;consumption\;\;{(m}^{3})}{Number\;\;of\;\;workers}$	34.87	43.63	50.92	62.56	79.90	53.69
$\frac{Average\;\;water\;\;consumption\;\;{(m}^{3})}{Number\;\;of\;\;beds}$	94.71	167.29	198.02	224.73	277.72	195.85

**Table 6:** Annual average consumption of water given by EMAS.

$\frac{Average\;\;water\;\;consumption\;\;{(m}^{3})}{Number\;\;of\;\;beds}$	HCNB 1	194
	HCNB 2	197
	HCNB 3	200
	HCNB 4	203

## Nomenclature

*A*: Value of the built area in a hospital, m^2^
HDDY: Heating degrees-day year, °C
NB: Total number of beds in a hospital
NW: Number of workers in a hospital
*T*_max_: Maximum daily temperature, °C
*T*_min_: Minimum daily temperature, °C
WC: Average annual consumption of CWHC, m^3^
*X*_*c*_: Logical coefficient.
